# A set-theoretic configurational analysis of sports event policy change: insights from Shanghai

**DOI:** 10.3389/fphys.2026.1748810

**Published:** 2026-04-10

**Authors:** Chengfeng Zhang, Jun Qiang, Linhua Chen

**Affiliations:** 1 Department of Physical Education, Shanghai University of Engineering Science, Shanghai, China; 2 Olympic Strategy Research Institute, Beijing Sport University, Beijing, China; 3 School of Sports Science and Engineering, East China University of Science and Technology, Shanghai, China

**Keywords:** Multiple streams framework, policy change, qualitative comparative analysis, sport policy, sports events

## Abstract

**Introduction:**

Grounded in the multiple streams framework, this study explores the configurations of political, policy, and problem stream factors that drive policy change in sports events.

**Methods:**

A crisp-set qualitative comparative analysis (csQCA) was conducted on 33 sports event policies enacted in Shanghai, China, from 2016 to 2022.

**Results:**

Four causal configurations leading to policy change were identified. Further classification revealed two distinct pathways: one driven by specific problems, and another shaped by the combined effects of political and policy factors.

**Discussion:**

This research advances understanding of the causal logic underlying sports event policy change and highlights the combined role of core multiple streams factors, thereby improving the rationality of practical policymaking.

## 1 Introduction

Sports events across various scales have become integral to contemporary destination management strategies, widely recognized for their capacity to stimulate socio-economic development, enhance tourism appeal, and elevate international visibility (Andersson et al., 2021; Chappelet, 2012; Gholipour et al., 2020; Knott et al., 2015). Governments and destination marketing organizations globally increasingly prioritize hosting major sports events, acknowledging their potential to foster urban transformation, cultural exchange, and infrastructural advancements (Leopkey et al., 2010). To fully leverage the positive externalities of sports events on destination development, most cities worldwide actively choose to formulate sports event policies, aiming to introduce and upgrade policy tools to enhance the contribution of sports events to urban development (Merkel and Kim, 2011; Whitford, 2009; Yu et al., 2018). However, the effectiveness of these policies determines whether sports events can promote high-quality development in destinations (Whitford, 2009). Among these factors, fully understanding the drivers of policy change is a critical component of crafting effective policies (Mukherjee et al., 2021). New institutional economics posits that policy change is a process where one policy is replaced by a more efficient alternative, which can also be interpreted as policy innovation (Greif and Laitin, 2004). Identifying the factors influencing policy change—that is, clarifying the conditions and forces that either drive or constrain policy change—can provide valuable insights for policymaking, facilitate the upgrading of policy tools, and enhance policy precision (Kay, 2017). Therefore, studying the factors influencing the change of sports event policies holds significant practical importance.

Although there is limited literature systematically integrating policy change theory into the study of sports event policies, some studies have addressed aspects of sports event policy changes. Existing research has concentrated on three main areas: performance evaluation, change trajectories, and influencing factors. Regarding performance evaluation, the absence of policy instruments is a prominent issue. For instance, Australian local governments have been criticized for producing hollow content and lacking long-term planning when drafting sports-event policies (Whitford, 2009), while the Swiss federal government has failed to provide systematic policy support for sports events (Leopkey et al., 2010). Goal-orientation bias is another salient problem: Scotland’s sports-event policies, for example, omit any reference to mass-sport participation (Meir et al., 2024). In terms of policy-change trajectories, strategies differ across countries owing to distinct national contexts. At the central level, China has shifted its policy emphasis from “government-run events” to “joint government–market delivery” (Zheng et al., 2018). Locally, Shanghai has explicitly moved from “building a first-class Asian sports city” to “creating a globally renowned sports city” (Yu et al., 2018). South Korea’s bid policy for the PyeongChang Winter Olympics regressed from a “joint North–South bid” to a “sole Korean bid” (Merkel and Kim, 2011), whereas Canada’s federal policy evolved from an early focus on the federal role and procedural standardization to a later emphasis on leveraging event legacies (McCloy and Thibault, 2013).With respect to influencing factors, emergent issues in event development (McCloy, 2009; Wang, 2016) and critical event windows (Chen et al., 2021) serve as key triggers for policy change. Nevertheless, the extant literature has not yet employed policy-change theories systematically to offer mechanistic explanations of the factors driving sports-event policy change, thereby leaving ample theoretical space for this study to deepen the analysis.

Broadly, current research exhibits the following gaps: 1) Existing studies have confined their conclusions to empirical generalizations, failing to embed “the drivers of sports event policy change” within a systematic policy-change theoretical framework for comprehensive analysis, and thus have been unable to accurately identify the full spectrum of factors driving policy change; 2) Existing studies have focused on analyzing policy texts, with few empirically testing research propositions through quantitative methods; 3) Few studies have adopted a nonlinear perspective to explain the configurational effects of determinants of sports event policy change. Against this research backdrop, this paper takes the sports-event policies promulgated by Shanghai, China as the research object, with the Multiple Streams Framework as the theoretical foundation and Qualitative Comparative Analysis (QCA) as the research method, to explore the factors influencing the change of Shanghai’s sports-event policy and its configurational paths, and further provide policymakers with actionable insights to translate sports event policies into effective levers of destination competitiveness. To achieve the above central research aim, the specific research questions/objectives of this study are formulated as follows:

1. What are the key influencing factors driving the change of Shanghai’s sports event policy in the context of urban development and global sports event industry evolution?

2. How do these key factors interact with each other to form configurational paths for the change of Shanghai’s sports event policy?

3. What actionable policy insights can be extracted from the configurational paths of Shanghai’s sports event policy change to optimize sports event policies and enhance destination competitiveness?

## 2 Literature review and analytical framework

### 2.1 Policy change

Policy change is a central focus in public policy research, with three predominant classical theories (McBeth et al., 2007): the Advocacy Coalition Framework, Punctuated Equilibrium Theory, and the Multiple Streams Framework. Originating from the work of Sabatier and colleagues (Sabatier and Jenkins-Smith, 1993), the Advocacy Coalition Framework (ACF) posits that policy change arises from three interrelated factors: (1) interactions among competing coalitions within a policy subsystem, (2) external perturbations such as socioeconomic shifts, public opinion trends, or changes in dominant coalitions, and (3) stable system parameters. A policy subsystem encompasses diverse actors—including government agencies, scholars, and civil society organizations—united by shared belief systems into distinct coalitions. The theory has found extensive application in analyzing elite sport policy (Chen, 2015; Green and Houlihan, 2004), serving as a framework to elucidate the determinants shaping their formulation. The Punctuated Equilibrium Theory (PET) (Baumgartner and Jones, 1993), employs a macro-level analytical lens to identify episodic shifts between policy stability and rapid change. This framework posits that policy systems oscillate between two states: long-term equilibrium (characterized by incremental adjustments) and short-term punctuation (marked by abrupt, transformative shifts). Key drivers of these transitions include shifts in policy image, institutional policy venues, the stability of policy monopolies, and the catalytic role of focusing events. The theory has subsequently been employed to elucidate the processes of policy change in both sport-related concussion (Quirion, 2017) and school physical education (Lindsey, 2020). The Multiple Streams Framework (MSF), developed by John Kingdon (Kingdon, 1984) and rooted in the Garbage Can Model (Cohen et al., 1972), identifies three interdependent factors influencing policy change: the problem stream (focusing shifts in critical indicators, focal events, and feedback on existing policies), the policy stream (consisting of policy proposals, solutions and academic recommendations polished and debated by policy communities including bureaucrats, interest groups, and domain-specific academics), and the political stream (reflecting shifts in public opinion, governmental administrative priorities, partisan alignments and institutional structural adjustments). Besides the three core streams, two pivotal supplementary elements of the MSF are the policy window and policy entrepreneurs: the policy window refers to a brief critical opportunity for the coupling of the three streams to promote policy change, while policy entrepreneurs are actors who leverage resources and strategic behaviors to facilitate the coupling of the three streams and the opening of policy windows. The theory has been widely applied to the analysis of sports policy. For example, the analysis of the dynamics and pathways of elite sports policy change (Zheng, 2017; Zheng and Liu, 2020), and determinants of school sports policy change (Houlihan and Green, 2006; Kristiansen and Houlihan, 2017; Reid and Thorburn, 2011). However, there are few studies systematically analyzing sports event policies within the framework of policy change theory at this stage. Addressing these research gaps, this paper employs the Multiple Streams Framework as its theoretical foundation to examine the sports-event policies promulgated by Shanghai, China.

### 2.2 Analytical framework

**Figure 1:**
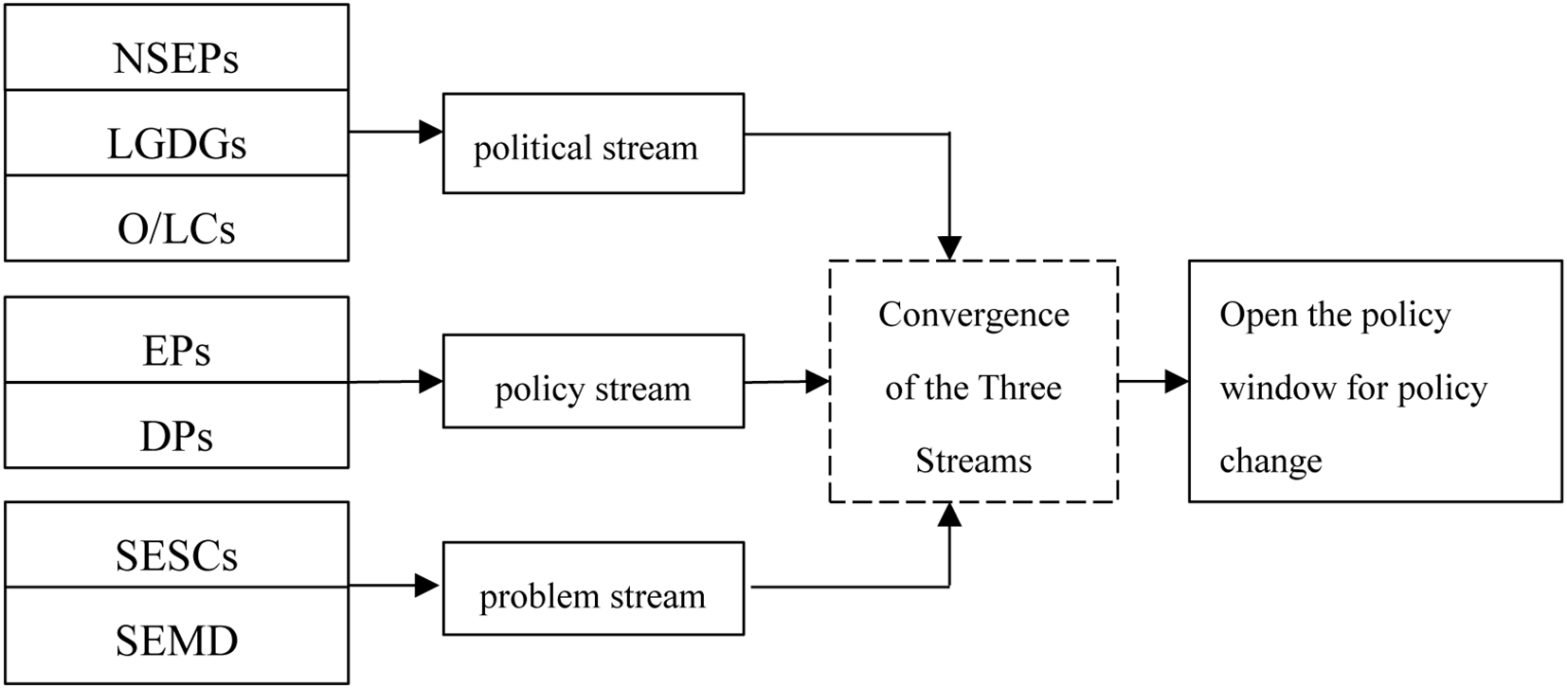
Analytical framework for the study. NSEPs, National sports event policies; LGDGs, Local government development goals; O/LCs, Organizational reforms or changes in leadership; Eps, Experts’ perspectives DPs, Proposals from deputies of the Shanghai Municipal People’s Congress SESCs, Sport event safety crises SEMD, Sport event market disorganization.

Multiple Streams Framework (MSF) has the advantage of simplicity, universality, and flexibility in analyzing the process of policy formulation (Jones et al., 2016; Weible and Schlager, 2016) and is able to explain the process of policy formation better enough to be applied to any field of policy analysis (Cairney and Jones, 2016). For the purposes of research, we take the MSF as the analytical framework to construct a theoretical analytical framework of determinants of sports event policy change in Shanghai (**Figure 1**).

In the problem stream, Kingdon (1984) posits that focusing events are critical catalysts for elevating issues to the policy agenda. Building on this theoretical foundation, this study examines the contextual drivers of policy formulation, revealing that focusing events—such as sport event safety crises (SESCs) and sport event market disorganization (SEMD)—have been found to be closely associated with regulatory interventions. Furthermore, existing research underscores that public safety concerns in sports events serve as a direct impetus for governmental policy action (Toohey and Taylor, 2008; Xuan, 2023). Moreover, Wang (2016) highlighted that non-compliant market practices in commercial and community sports events constitute a key driver of policy reforms within China’s sports event governance framework. Consequently, this study identifies SESCs and SEMD as critical factors within the problem-stream domain.

Within Kingdon’s (1984) multiple streams framework, policy recommendations generated by the policy community constitute the “policy broth” that informs agenda-setting and policy formulation. In the Chinese context, National People’s Congress (NPC) deputies and subject-matter experts form the core of this policy community. Existing scholarship emphasizes the instrumental role of NPC legislative proposals and academic research in shaping China’s sports policy change, particularly in bridging national strategic directives with localized implementation challenges (Hou et al., 2022; Wang et al., 2021). Consequently, this study identifies proposals from deputies of the Shanghai Municipal People’s Congress (DPs) and experts’ perspectives (EPs) as key influencing factors within the policy stream.

To refine the analysis of political stream dynamics, this research adopts modified Multiple Streams Framework. Zahariadis (1995) contends that the original political stream drivers—shifts in public sentiment, interest group activity, and administrative turnover—were conceptualized within the context of the U.S. separation-of-powers system. Therefore, reconceptualizing Kingdon’s political stream drivers—shifts in public sentiment, interest group dynamics, and administrative reshuffling—through the lens of a centralized governance system reveals their alignment with the ruling party’s strategic priorities.

In China’s political context, this study examines the political source stream’s influence on local sports event policymaking through the lens of the ruling party’s strategic orientation. National policies, serving as institutional embodiments of the ruling party’s governance philosophy, demonstrate pronounced vertical transmission effects within China’s political institutional framework. Specifically, the central government’s macro-level strategies and policy directives systematically shape subnational decision-making through institutionalized channels of political authority allocation and administrative implementation mechanisms (Sun and Guo, 2021). National sports event policies (NSEPs) carry demonstrable top-down political imperatives, forming a foundational institutional framework for municipal policymaking (Zheng, 2015; Zheng et al., 2018). Furthermore, local government development goals (LGDGs) constitute the concrete manifestation of the ruling party’s will, and both tourism-policy research and sports-industry-policy studies have explicitly affirmed that LGDGs exert a profound influence on local policy formulation (Bramwell and Lane, 2011; Zheng, 2014). In the case of Shanghai, China, the city has proposed the development goal of building an international event capital, and the realization of the goal requires the formulation of a series of sports event policy to be implemented, so LGDGs also exerts an influence on the change of local sports event policy. In addition, the will of the ruling party is affected by internal organizational reforms or changes in leadership (O/LCs), which in turn affects public policy formulation (Kingdon, 1984). Scholarly research demonstrates that shifts in key decision-making roles can trigger measurable recalibrations in policy trajectories, particularly within centralized governance systems where administrative continuity aligns closely with party priorities (Bao and Ma, 2018). Accordingly, this paper considers O/LCs, NSEPs, and LGDGs as motivating factors at the level of political flows.

## 3 Methods

This study uses a configurational perspective to explore the causal mechanisms of China’s local sports event policy changes, with Qualitative Comparative Analysis (QCA)—a set-theoretic comparative method that uncovers social phenomena’s causal complexity by identifying condition combinations leading to the same outcome, stressing equifinality and causal asymmetry—as the core method. QCA is ideal for multi-factor conjunctural complexity and small-to-medium-N research (Ragin, 2008). The study adopts csQCA, calibrating determinants as binary variables to identify core factors and compare causal asymmetry.

### 3.1 Explanation of research case location and sample selection

The reasons for choosing Shanghai as the research case location in this paper are specifically as follows: (1) Shanghai is the most active city in China in organizing international and domestic sports events. The official website of the Shanghai Municipal People’s Government shows that the number of international and domestic sports events to be held throughout the year 2024 reaches 178, and the number of international and domestic sports events to be held throughout the year 2025 is 171 (Chen, 2025). Shanghai has introduced a package of policies and measures to vigorously develop the sports event economy. (2) The reasons for the introduction of Shanghai’s sports event policies are complex. First, in order to build an international sport events capital, Shanghai has introduced a series of policy measures based on the development will of the local government. Secondly, in order to cope with the “aftermath of the abolition of the right to approve commercial and mass sports events”, Shanghai has duly introduced relevant rules and regulations to regulate the event market players. Third, in order to prevent the risk and safety management of sports events, Shanghai has issued a series of safety control documents for sports events. Overall, the cases of sports event policies introduced by Shanghai are in line with the theoretical sampling principles of similarity and heterogeneity for case selection in qualitative comparative analysis method.

This study employs various types of sports event policies issued since the “13th Five-Year Plan for the Reform and Development of Sports in Shanghai” (2016-2022) as the object of study (**Supplementary Material Table 1**). In terms of the types of policies, encompassing both specialized sports event policies issued by the Shanghai Municipal Sports Bureau and provincial-level directives promulgated by the Shanghai Municipal Government. To ensure methodological consistency with crisp-set QCA (csQCA) requirements, co-issued policies involving multiple departments (e.g., Cases 3, 15, 29) are categorized under the lead agency’s jurisdiction. The case selection strategy incorporates supplementary sports industry policies containing explicit event development provisions (Cases 1, 3, 6, 12, 14, 15, 17, 20, 25, 26, 29, 32, 33) to enhance case diversity while maintaining thematic relevance. All policies were sourced exclusively from the official portal of the Shanghai Municipal Sports Bureau, ensuring procedural transparency in text retrieval.

### 3.2 Variable selection and operationalization

Following csQCA protocol, all variables receive dichotomous coding (1 = presence, 0 = absence), as detailed in **Table 1**.

**Table 1:** The assignment to the outcome variable and the condition variable.

Variable	Variable type	Data weight	Assignment value	Notes
NSEPs	Presence of NSEPs influence	21.21%	1	Condition variable
	Absence of NSEPs influence	78.79%	0	
LGDGs	Presence of LGDGs influence	69.70%	1	
	Absence of LGDGs influence	30.30%	0	
O/LCs	Presence of O/LCs	6.06%	1	
	Absence of O/LCs	93.94%	0	
EPs	Presence of EPs influence	69.70%	1	
	Absence of EPs influence	30.30%	0	
DPs	Presence of DPs influence	21.21%	1	
	Absence of DPs influence	78.79%	0	
SESCs	Presence of SESCs	18.18%	1	
	Absence of SESCs	81.82%	0	
SEMD	Presence of SEMD	12.12%	1	
	Absence of SEMD	87.88%	0	
Policy Change	Policy at Shanghai Municipal Sports Bureau	81.82%	1	Outcome variable
	Policy at provincial level	18.18%	0	

NSEPs, National sports event policies; LGDGs, Local government development goals; O/LCs, Organizational reforms or changes in leadership; Eps, Experts’ perspectives DPs, Proposals from deputies of the Shanghai Municipal People’s Congress SESCs, Sport event safety crises SEMD, Sport event market disorganization.

#### 3.2.1 Variable selection and operationalization

Policy Change is defined as the adjustment, modification, or fundamental transformation of public policy objectives, content, instruments, or implementation. This concept represents the policy system’s response to shifts in the external environment, internal pressures, or evolving societal perceptions, reflecting dynamic interactions among social needs, political dynamics, technological advancements, and value systems (Kingdon, 1984). In tourism policy research, Sun and Guo (2021) operationalized policy change as a binary outcome variable (1 or 0) using a bifurcation framework. Policies enacted by municipal-level authorities (i.e., administrative bodies superior to local Tourism Bureaus) were coded as 1, while those introduced by local Tourism Bureaus were coded as 0. Similarly, Bao and Ma (2018) applied this binary coding method to analyze environmental protection policy changes in Lanzhou City, assigning a value of 1 to policies formulated by the Urban Management Committee or higher authorities and 0 to those issued by the Comprehensive Administrative Enforcement Bureau of Urban Management. Building on this methodological approach, the present study examines tournament policies issued by the Shanghai Municipal Sports Bureau. Policies enacted by the Bureau itself are coded as 1, while those promulgated by provincial-level authorities (administrative bodies superior to the Bureau) are coded as 0.

#### 3.2.2 Conditional variable

SESCs: The sport event safety crises play a significant role in shaping policy. SESCs is defined as a critical emergency that poses a severe threat to public safety for participants (athletes, spectators, and staff), event operations, or broader societal stability. Such crises may arise from terrorist attacks, natural disasters, crowd stampedes, infrastructure failures, public health emergencies, or systemic management failures during the preparation, staging, or closing stages of a sports event (Leopkey and Parent, 2009; Toohey and Taylor, 2008). Major safety incidents and public safety crises have been identified as critical drivers of event policy reform (Giulianotti and Klauser, 2010). Consequently, the occurrence of an event safety crisis was coded as 1, while its absence was coded as 0.

SEMD (market): Irregular behaviors in sport events plays a significant role in shaping policy (Wang, 2016). SEMD primarily refers to the regulatory vacuum that emerged following the Chinese government’s abolition of its authority to approve sports events in 2014. Some local governments subsequently failed to implement effective oversight during and after the events, leading to various adverse consequences in the event organization process. Specifically, event organizers exhibited chaotic management that resulted in poor service quality, engaged in false advertising to attract participants, exploited events for gambling and fraud, failed to fulfill their contractual obligations, and falsely claimed government sponsorship of their events. In response, the Chinese government has promulgated numerous policies to regulate market behavior (Wei and Liu, 2018). Based on this, this study categorizes the policies listed in **Supplementary Material Table 1** into two groups. The first group consists of policies specifically targeting the disorder in Shanghai’s sports event market, assigned a value of 1. The second group comprises policies not directly addressing such market disorder, assigned a value of 0.

EPs: The experts’ perspectives play a significant role in shaping policy. EPs primarily encompass the perspectives of sports event experts and scholars, which serve as influential factors shaping the development of policy. Drawing on Kingdon’s (1984) framework, scholars disseminate their academic insights through publications, writings, and public speaking, thereby exerting influence on public policy formation. Consequently, this study views the academic perspectives of experts and scholars specializing in Shanghai’s sports events as critical drivers of event-policy development. The Sports Event Research Center at Shanghai Sport University emerges as a pivotal think tank dedicated to advancing the development of Shanghai’s sports events. Its primary mission is to provide intellectual support for Shanghai’s ambition to establish itself as an international event hub. Additionally, the Policy and Regulation Department of the Shanghai Municipal Bureau of Sports includes representatives from the think tank’s political sector, who are well-acquainted with the think tank’s senior experts and have cultivated a relatively close professional network. Since its inception, the think tank has been instrumental in drafting a series of policies governing Shanghai’s sports events, as well as the city’s and national-level “five-year plans” for sports development. Therefore, the identification of expert academic views in this study is grounded in the academic perspectives of the members of the Sports Event Research Center at Shanghai Sport University. This paper examines the academic publications and writings authored by the experts of the Sports Event Research Center at Shanghai Sport University, as well as the viewpoints they express in public media forums. It organizes their core perspectives on fostering the development of Shanghai’s sports events (**Supplementary Material Table 2**). The presence of relevant experts’ academic views in the policy is assigned a value of 1, while their absence is assigned a value of 0.

DPs: The proposals from the deputies of Shanghai Municipal People’s Congress play a significant role in shaping policy, particularly in the context of Shanghai’s sports events. In Western political systems, parliamentarians serve as key members of the policy community, exerting substantial influence over the formulation of public policies (Kingdon, 1984). Similarly, in China’s political framework, the proposals of NPC deputies constitute one of the critical factors influencing policy drafting. Elected through democratic processes, NPC deputies represent the interests and aspirations of the populace, impacting policy-making through their participation in legislative sessions and submission of motions. Existing literature on China’s public sports policy frequently highlights instances where NPC deputies’ proposals have catalyzed the evolution of the nation’s sports policy framework (Wang et al., 2021). In this study, we conducted a search on the official website of the Shanghai Municipal Bureau of Sports to retrieve publicly available outcomes of relevant proposals and systematically organized their content (**Supplementary Material Table 3**). The incorporation of NPC deputies’ proposal viewpoints into policy demonstrates their pivotal role in shaping governmental decisions.

NSEPs: National sports event policies play a significant role in shaping policy. NSEPs refers to the influence of sports event policies issued at the national level (central government) on the development of sports events in Shanghai. Within China’s political context, national policies developed and implemented by the central government are distinguished by their national scope, authority, and guiding nature. The formulation and implementation of local policies must adhere to the overarching framework and requirements of national policies and cannot contradict them. Local policy drafting autonomy essentially functions as a “limited experiment” within the centralized control framework (Heilmann, 2008). Consequently, national policies impose distinct constraints on the development and execution of local policies. In the realm of sports event policy, local policy drafting similarly faces these constraints (Zheng, 2015; Zheng et al., 2018). Based on this, this study assigns a value of 1 to the presence of a national event policy and a value of 0 to its absence in relation to the content of the case policy (**Supplementary Material Table 4**).

LGDGs refer to the influence of local governments’ development goals on the introduction of sports event policies. Specifically, the Shanghai Municipal People’s Government explicitly set the goal of building Shanghai into a world-class capital of international sports events in 2015 (Yu et al., 2018). Guided by this overarching goal, successive Shanghai governments have introduced a range of policy tools tailored to sports event development. Through an analysis of the specific content of case policies, all policies are categorized into two groups: (1) proactive policy tools designed by Shanghai municipal governments to support the construction of an international events capital, which are assigned a value of 1, and (2) reactive policies formulated to address unforeseen challenges during the development process, which are assigned a value of 0.

O/LCs refer to the impact of personnel adjustments or organizational restructuring within institutions responsible for managing sports event development on the formulation of Shanghai’s sports event policies. According to multiple streams theory (Kingdon, 1984), personnel changes or organizational shifts can lead to policy changes, emphasizing the significance of such dynamics in the policy-making process. Additionally, research has shown that local officials transferring across regions tend to introduce policies from their previous postings, contributing to policy changes due to personnel adjustments (Zhu and Zhang, 2016). Through a detailed analysis of the case policies, this study categorizes all policies into two groups: (1) those influenced by organizational or leadership changes, assigned a value of 1, and (2) those unrelated to such changes, assigned a value of 0.

## 4 Results

### 4.1 Necessity analysis of individual conditions

According to the analytical steps of the qualitative comparative analysis method, it is necessary to test the necessity of each antecedent condition individually before carrying out the group analysis. In this study, the necessity test of all antecedent conditions is conducted using fsQCA3.0 software. As shown in **Table 2**, the consistency test level of all antecedent conditions is below 0.9, indicating that individual antecedent conditions are not necessary for the existence of the results. In other words, the introduction of Shanghai’s sports event policy (Policy at Shanghai Municipal Sports Bureau) is influenced by a variety of factors.

**Table 2:** Necessary analysis result.

Condition	Consistency	Coverage
NSEPs	0.148148	0.666667
~ NSEPs	0.851852	0.851852
LGDGs	0.666667	0.782609
~ LGDGs	0.333333	0.900000
O/LCs	0.074074	1.000000
~ O/LCs	0.825926	0.806452
EPs	0.666667	0.782609
~ EPs	0.333333	0.900000
DPs	0.074074	0.285714
~ DPs	0.825926	0.961538
SESCs	0.222222	1.000000
~SESCs	0.777778	0.777778
SEMD	0.111111	0.750000
~SEMD	0.888889	0.827586

### 4.2 Sufficiency analysis of conditional groupings

Sufficiency analysis of conditional groupings aims to test whether a set of multiple antecedent conditions forming a grouping is a subset of the outcome combination. This analysis seeks to explore the sufficiency of combinations of multiple different antecedent conditions in explaining the existence of the outcome. In conducting sufficiency analysis, researchers must determine the criteria for setting raw consistency and frequency thresholds based on the specific study. The minimum level of raw consistency should not fall below 0.75, with the software typically defaulting to a value of 0.8 (Schneider and Wagemann, 2012). Frequency threshold levels should be adjusted according to the specific sample size. For large samples, thresholds are typically set greater than 1, while small and medium samples generally use a threshold of 1. A widely accepted guideline for frequency threshold settings is that at least 75% of the total sample size should be retained (Schneider and Wagemann, 2012). In this study, the raw consistency level and frequency count threshold were set to their default values of 0.8 and 1, respectively. In addition, due to the paucity of research in existing literature on the influencing factors of sports event policy change, this paper adopts the default labeling for counterfactual analysis during software operation. Specifically, it assumes that the presence or absence of a single antecedent condition can serve as the cause of policy change in Shanghai’s sports event policy.

This study employs fsQCA3.0 software to analyze the sports event policies issued by the Shanghai Municipal Sports Bureau, generating three types of solutions: complex, simple, and intermediate. Following international best practices, the intermediate solution is compared with the simple solution to report the findings. The results are presented using symbolic expressions (Fiss, 2011) (**Table 3**), where a solid circle (●) indicates the presence of a condition, a forked circle (⊗) indicates its absence, and an empty space indicates uncertainty regarding the condition’s presence. The large circle represents the core condition, which appears in both the intermediate and simple solutions, while the small circle denotes the auxiliary condition, exclusive to the intermediate solution. The intermediate solution comprises four grouping solutions, with each achieving a consistency level of 0.8 at the individual grouping level. Among these groupings, grouping 3 exhibits the highest levels of unique and original coverage, making it the most empirically relevant grouping. Overall, the consistency level of the four histogram solutions exceeds 0.8, and the total coverage is 1. Therefore, the four histograms collectively constitute a sufficient condition for policy change at the Shanghai Municipal Sports Bureau level.

**Table 3:** Sufficiency analysis results.

Condition	Configurational analysis			
	Problem-driven		Multifactor superposition	
	1	2	3	4
NSEPs		⊗	⊗	●
LGDGs	⊗	⊗	●	●
O/LCs	⊗	⊗	⊗	●
EP	⊗	⊗	●	●
DPs	⊗	⊗	⊗	●
SESCs	●	⊗	⊗	⊗
SEMD	⊗	●	⊗	⊗
Consistency	1	1	1	1
Raw coverage	0.222222	0.111111	0.592593	0.0740741
Unique coverage	0.222222	0.111111	0.592593	0.0740741
Solution consistency	1			
Solution coverage	1			

a solid circle (●) indicates the presence of a condition, a forked circle (⊗) indicates its absence, and an empty space indicates uncertainty regarding the condition’s presence.

### 4.3 Robustness testing

Robustness testing of the results is an essential component of the qualitative comparative analysis (QCA) method. In this study, the reliability of the findings was assessed by increasing the consistency level threshold from 0.8 to 0.85. Specifically, a more stringent threshold was applied to test the robustness of the results. The analysis revealed that the intermediate solution groupings derived from the analysis remain fully consistent with the pre-adjustment groupings. Additionally, the consistency and coverage levels after adjustment remain at a high standard, indicating that the findings of this study are robust (**Supplementary Material Table 5**).

## 5 Discussion

To identify the determinants of local sports event policy change, this study analyzes 33 Shanghai cases through the lens of the multiple streams framework. Using crisp-set qualitative comparative analysis, the results reveal four distinct pathways driving policy changes (**Figure 1**): (1) SESCs, (2) SEMD, (3) alignment of LGDGs with EPs, and (4) the interplay of NSEPs, LGDGs, O/LCs, EPs, and DPs. Based on these pathways, the study categorizes the configurations into two types. Pathways 1 and 2 share similar issue-driven mechanisms rooted in problem stream factors and are thus classified as issue-driven. Pathways 3 and 4, however, reflect multi-factor superimposed driving, arising from the convergence of political stream factors (Pathway 3) and policy stream factors (Pathway 4). Both pathways demonstrate multi-factor interactions that collectively trigger policy change.

Configuration 1 demonstrates that, in the absence of core conditions (DPs, SEMD) and marginal conditions (LGDGs, O/LCs, EPs), SESCs emerges as a driving factor for changes in relevant policies. Configuration 1 encompasses six cases (Cases 4, 9, 10, 11, 16, and 18), all of which are directly related to sport event safety. This demonstrates that safety risks in sporting events constitute a significant factor driving the formulation of sports event policies. Although existing studies have alluded to this perspective (Giulianotti and Klauser, 2010; Toohey and Taylor, 2008), this study represents the first quantitative verification of the causal relationship between the two. This finding also serves as a crucial reminder for policymakers to proactively identify safety risks associated with sporting events.

Among the analyzed cases, the policy contents of Cases 10, 16, and 18 are closely aligned with public health safety, sharing a common backdrop tied to the COVID-19 pandemic. The pandemic presented significant challenges to the organization of major international and domestic sporting events in Shanghai. To safeguard public health and ensure societal stability and orderly operations, the Shanghai Municipal Bureau of Sports issued a series of guidelines aimed at preventing public health incidents during events. Examining the temporal trajectory of policy changes, Case 18 was introduced the earliest, followed by Case 16, and then Case 10. Furthermore, in terms of policy themes, the three policies represent a continuum focused on safety, epidemic prevention, and control. Each policy iteration builds upon revisions of the preceding policy text, with emerging situations and challenges during the epidemic serving as the primary drivers for policy updates. The progression from the original version to the third edition underscores the Shanghai Municipal Bureau of Physical Education’s emphasis on the dynamic adaptability of policies during formulation. This iterative approach demonstrates the bureau’s proficiency in timely and context-specific adjustments to policy initiatives, aligning them with the evolving phases of the epidemic’s development and problem dynamics.

Cases 9 and 11 represent a continuum of policies aimed at preventing traditional safety issues in races. These policies were introduced in response to the “2021 Baiyin marathon trail-running tragedy in Gansu Province, China,” which occurred on May 22, 2021.The accident resulted in the deaths of 21 participants due to adverse weather conditions and human error on the part of the race organizers. This tragedy prompted the General Administration of Sport of China to investigate the fatalities and intensify its focus on the safety supervision of commercial and mass sports events. Consequently, the central government convened the “National Conference on Strengthening the Safety Management of Events in the Sports System.” To implement the conference’s directives, provinces and cities, including Shanghai, issued localized event safety policies. Specifically, the Shanghai Municipal Bureau of Sports released the Case 11. To address local conditions and advance event safety rectification efforts, the bureau issued the Case 9 just over two weeks after the initial policy. Both policies were driven by unexpected safety incidents at events, underscoring the importance of timely policy adjustments in response to tragic occurrences. Case 11 was issued on June 3, 2021, just one day after the “National Sports System Conference on Strengthening Event Safety Management” was held on May 23, 2021. This demonstrates the swift responsiveness of the Shanghai Municipal Bureau of Sports during the policy formulation process. Comparing the issuance dates of Cases 11 and 9 further highlights the rapidity of the policy response. Additionally, a supplementary notice for Case 9 was issued on June 11, 2021, to ensure the policy initiative aligned with the local situation. This iterative approach underscores the bureau’s commitment to timely and context-specific adjustments in response to emerging challenges.

In addition, the Case 4 represents a comprehensive policy addressing both traditional and non-traditional risks associated with sport events safety management. This policy serves as a complementary measure to the previously mentioned five policies and reflects the Shanghai Municipal Sports Bureau’s ongoing commitment to regulating event safety. With the normalization of the COVID-19 pandemic and the persistent presence of traditional risks, the convergence of these dual challenges necessitates enhanced safety management policies for sports events at the local administrative level.

Configuration 2 demonstrates that, in the absence of NSEPs, LGDGs, O/LCs, EPs, DPs, and marginal conditions related to SESCs, SEMD emerges as the key influencing factor driving the issuance of relevant policies. The policies corresponding to Configuration 2 are Cases 5, 28, and 31, all of which are regulatory documents aimed at governing the behavior of the sports event market. Unruly behavior during tournament organization serves as the primary catalyst for policy issuance. The results demonstrate that SEMD can directly prompt policy interventions, aligning with prior scholarship that identifies such disruptions as a critical catalyst for regulatory adjustments (Wang, 2016). However, unlike existing studies—which predominantly rely on theoretical assertions—this research employs a comparative multi-case analysis grounded in the multiple streams framework. This approach empirically validates market disorder as a discrete driver of policy formulation, addressing a key gap in prior work by systematically tracing causal mechanisms rather than merely positing correlations. The methodology further demonstrates methodological rigor through structured process tracing, advancing beyond speculative claims to establish replicable analytical pathways.

In terms of specific policies, the Shanghai Municipal Bureau of Sports issued the Case 31 in June 2017. This marked the first national-level guidance document on mass sports events and represented a significant initiative to innovate the governance of such events. Following the introduction of the State Council’s Document No. 46, which abolished the approval authority for commercial and mass sports events, two notable trends emerged: a rapid surge in the number of registered enterprises and events nationwide, and the lack of oversight leading to disorganization in commercial and mass events. These factors contributed to irregularities in event organization. For instance, enterprises exhibited varying capabilities in organizing tournaments, false advertising lured sponsors and spectators into purchasing tickets, and the absence of government support for commercial events resulted in reluctance from departments such as public security, firefighting, health, and transportation to assist in tournament operations.

Shanghai, as an active city in organizing commercial and mass sports events, has experienced the rapid exposure of numerous irregularities in tournament organization, leading to significant disorder in the sport event market. To strengthen the normalization of mass sports event organization, the Shanghai Municipal Bureau of Sports swiftly reacted and issued timely policies to intervene, despite the absence of a specialized regulatory system at the time. Case 28 and Case 5 are documents outlining the responsibilities of the Shanghai Municipal Bureau of Sports in tournament organization, demonstrating continuity and inheritance in their content. Case 28 was issued in July 2018, a key background factor for this policy was the lack of management over event naming and the unauthorized use of the Shanghai Municipal Bureau of Sports and Shanghai Sports Federation names by tournament contractors and co-organizers. To define the responsibilities of the Shanghai Municipal Bureau of Sports in tournament organization, this document was introduced. Building on Case 28, Case 5 was issued in January 2022. This policy represents a further refinement and supplementation of the earlier provisions, reflecting the bureau’s commitment to continuous improvement in policy development.

Configuration 3 demonstrates that, in the absence of core conditions (NSEPs, DPs) and peripheral conditions (O/LCs, SESCs, SEMD), peripheral conditions such as LGDGs and EPs play a significant role in driving policy changes. The results indicate that policy innovation is shaped by the synergistic interplay of LGDGs and EPs. Although existing studies have shown that LGDGs are a determinant the change of local policies (Bramwell and Lane, 2011; Zheng, 2014), and other research has indicated that EPs profoundly shape local policy-making (Hou et al., 2022; Wang et al., 2021), our study further reveals that the interaction between LGDGs and EPs jointly drives the change of local policies. The relationship between the two is not independent or mutually irrelevant. Moreover, for the first time, we empirically examine the impact of their interaction on the introduction of local sports event policies. Our research deepens the understanding of studies on the change of sports event policies.

Configuration 3 corresponds to 16 policies, specifically Cases 1, 2, 6, 7, 8, 12, 13, 19, 20, 22, 23, 24, 26, 27, 29, and 30. In terms of explanatory power, Configuration 3 represents the most dominant pathway influencing policy changes, accounting for the logic of change for the majority of the policies in the dataset. The introduction of these policies shares a common objective: to advance Shanghai’s development as an international tournament capital. To achieve this, the local sports administration has proactively introduced the aforementioned policies, which encompass substantive initiatives such as tournament information collection, tournament brand authentication, tournament impact assessment, tournament financial support, regional synergy in tournament organization, tournament system construction, tournament standardization development, and tournament cluster planning.

When compared to Configuration 1 and 2, the policies corresponding to Configuration 3 are not the result of passive problem-driven actions but rather proactive measures influenced by LGDGs (specifically, building an international event capital) and EPs. The interaction between the LGDGs of the Shanghai Municipal Sports Bureau and the EPs forms the driving force behind the introduction of these policies. This mutually reinforcing relationship arises from the collaboration between the sports administration and university think tanks. The Sports Event Research Center at Shanghai University of Sport stands out as a leading university think tank in China’s sports industry, with a long-standing involvement in shaping national and Yangtze River Delta region sports industry policies. The research findings of relevant experts have served as a foundational element (“policy primeval soup “) for sports event policies. However, not all research outcomes or academic perspectives are elevated to policy documents; only those initiatives that align with the development goal of establishing an international event capital and are endorsed by the Shanghai Municipal Sports Bureau gain entry into the policy-making process.

Configuration 4 demonstrates that, even in the absence of marginal conditions such as SESCs or SEMD, core conditions (O/LCs) and marginal conditions (e.g., NSEPs, LGDGs, EPs, and DPs) remain the drivers influencing the Shanghai Municipal Bureau of Sports to formulate policies. This result demonstrates that policy changes emerge from the nonlinear interplay of multiple institutional factors. While prior research has identified NSEPs (Zheng, 2015; Zheng et al., 2018), LGDGs (Zheng, 2014), DPs (Hou et al., 2022; Wang et al., 2021), as discrete drivers of policy reform, existing analyses remain constrained by linear causal frameworks. Such approaches overlook the synergistic effects of factor interactions—a critical gap addressed here through systems-based analytical modeling. By adopting a complexity theory lens, this research reveals how dynamic, non-additive relationships between variables reconfigure policy landscapes, marking a methodological advance beyond conventional reductionist paradigms. Furthermore, for the first time, we identify O/LCs as one of the determinants in policy change.

The corresponding cases in Configuration 4 are cases 3 and 15, both of which focus on advancing the collaborative efforts of the Yangtze River Delta region (comprising Jiangsu, Zhejiang, Anhui, and Shanghai) in organizing regional tournaments and collectively bidding for major international sporting events. Regional tournament co-organization serves as a critical component of the Yangtze River Delta’s integrated sports development strategy, rooted in the shared developmental aspirations of the three provinces and one city, while also being shaped by NSEPs, EPs, and DPs.

Specifically, the first factor is influenced by the NSEPs aspect. In December 2019, the State Council issued the Outline of the Plan for the Integrated Development of the Yangtze River Delta Region. According to the policy’s overall deployment, in October 2020, the three provinces and one city in the Yangtze River Delta jointly issued the “Opinions on the High-Quality Development of the Integration of Sports in the Yangtze River Delta Region” (Case 15). This policy explicitly outlined the Yangtze River Delta’s integrated approach to tournament planning. To further clarify the content and initiatives for the integrated development of the regional sports industry, in January 2022, the three provinces and one city also jointly issued the “Yangtze River Delta Regional Sports Industry Integration and Development Plan (2021-2025)” (Case 3), which provides a more detailed framework and design for the integrated operation of tournaments in the Yangtze River Delta.

Secondly, the initiative was significantly influenced by the EPs and the DPs. Prior to the introduction of Cases 15 and 3, the concept of promoting the synergistic organization of tournaments in the Yangtze River Delta was repeatedly mentioned in the studies of relevant experts and the DPs.

Thirdly, the initiative was influenced by the emergence of a new organizational structure. Among the many influencing factors, the organizational structure established to promote the integrated development of sports in the Yangtze River Delta plays the most crucial role. During the process of promoting the integrated development of sports in the Yangtze River Delta, to synergize the development interests of all parties and align regional development goals, cross-regional organizations such as the Yangtze River Delta Regional Sports Industry Collaboration Association, the Joint Meeting on Sports Integration in the Yangtze River Delta, the Yangtze River Delta Sports Integration Office, and the Yangtze River Delta Sports Industry Alliance have been established sequentially. The existence of these organizations, on the one hand, elevates the informal expression of policy stakeholders’ demands into formal policy documents, and on the other hand, based on the principle of maximizing common interests, facilitates consultations on the event development goals and development initiatives of different regions. Therefore, the emergence of cross-regional organizations has played a dominant role in the policy of regional coordinated tournament running.

## 6 Conclusion and implications

This study examines the determinants of Shanghai’s sports event policies change through the lens of the multiple streams framework. Utilizing csQCA, we demonstrate that the change of policies is contingent upon the interplay of multiple factors. At the problem stream level, SESCs catalyze the formulation of related policies. Similarly, SEMD at the problem stream level drives policy issuance. At the political stream level, LGDGs, coupled with EPs at the policy stream level, constitute the most significant determinant of policy change. Lastly, a combination of NSEPs, LGDGs, and O/LCs at the political stream level, alongside EPs and DPs at the policy stream level, collectively facilitate policy introduction. Furthermore, the influence paths can be categorized into two types: problem-driven and multifactor superposition configurations. Broadly, this study makes three key contributions to the field of sports policy research. First, we ground our work in Multiple Streams Theory (Kingdon, 1984) to construct a rigorous analytical framework for explaining policy changes in sports events, thereby significantly extending the theory’s applicability within the sports policy domain. Second, whereas prior research has merely offered empirical summaries of the determinants of sports event policy change (Chen et al., 2021; McCloy, 2009; Wang, 2016), we are the first to construct a clear and rigorous analytical framework that systematically identifies and integrates the core drivers of China’s sports-event policy shifts, thereby effectively closing a critical gap in the literature. Third, methodologically, we pioneer the use of Qualitative Comparative Analysis (QCA), leveraging set-theoretic logic to examine the configurational causes of policy evolution, thus offering a novel methodological lens for future research on sports event policy.

From a policy intervention perspective on enhancing the quality development of sports events, our study offers valuable practical insights for cities globally to harness sports events for destination development. First, proactively monitor emerging issues in sports-event practice and continuously refine event-policy formulation. Specifically, 1) establish an information-gathering system for event-related problems. Before any competition, convene a joint stakeholder meeting to anticipate and analyze potential challenges. Require every organizing committee to add a mandatory “event-problem statement” section in its post-event summary report. 2) Increase the speed of policy response. Problems that hinder sports-event development can erupt suddenly and produce immediate, serious negative effects. Upon identifying such problems, the local sports-event regulator must immediately assemble experts and scholars to formulate targeted policies. 3) Adjust policy instruments on demand. Issues in sports events evolve continuously; their negative impacts may fade under policy intervention, ushering in a new development phase. During this new cycle, the original measures may no longer suit the changed circumstances and must be updated accordingly.

Second, local sports administrative departments should deepen collaboration with university-based sports-event think tanks to co-create development initiatives. On the one hand, efforts should be made to strengthen the top-level design of development planning for local sports events. To develop the sports event economy, local sports administrative departments should formulate long-term and clear development goals. Policies issued for sports events should be compatible with the overall goals and top-level design, and serve as specific tools to achieve such top-level design. For example, after Shanghai put forward the goal of building an “International Sports Events Capital”, its subsequent policies and measures were formulated around this top-level design, including sports event information collection, event brand certification, impact evaluation, financial support, regional cooperation in hosting events, construction of the event system, standardization of events, and planning of sports event clusters. On the other hand, cooperation with university-based think tanks for sports events should be enhanced. Guided by the long-term planning of local sports events, local sports administrative departments should jointly build research think tanks for sports events with local universities, actively build a platform for policy communities to express their views, and give full play to the wisdom and strength of policy communities. By setting up decision-making consultation research projects to encourage university scholars to participate in event policy research, establishing a database of sports event experts, and enhancing dialogue between sports administrative departments and event experts, useful views from policy communities can be fully absorbed. This will further expand participation and transparency in policy-making, and improve its rationality, operability and foresight.

## 7 Limitation and future research

This study examines the determinants of sports event policy change in Shanghai, focusing primarily on policies issued by the Shanghai Municipal Bureau of Sports. A notable limitation of this research design is its exclusion of provincial-level policies. While this omission stems partly from objective constraints, the primary challenge lies in the limited sample size of provincial policies. Between December 2016 and March 2022 – corresponding to the period after the release of the 13th Five-Year Plan for the Reform and Development of Shanghai Sports – only six provincial-level policies were identified. Furthermore, policies issued by the Shanghai Municipal People’s Government or its General Office remain relatively limited, with the majority still originating from the Municipal Sports Bureau. Given this scarcity of provincial-level cases, conducting a robust analysis of their variation drivers proves methodologically untenable. Future research should extend the temporal scope to compile a robust sample (minimum n=10) of provincial-level policies, enabling dedicated analysis of their variation drivers as the primary investigative focus.

## Data Availability

The original contributions presented in the study are included in the article/**Supplementary Material**. Further inquiries can be directed to the corresponding authors.
