# Supplementary material for: A set-theoretic configurational analysis of sports event policy change: insights from Shanghai
Source: Front Physiol. 2026 Apr 10;17:1748810. doi: 10.3389/fphys.2026.1748810 (PMC13105456; doi:10.3389/fphys.2026.1748810)
Supplement: Supplementary file 1 [file Table1.docx]

**Table1. Case Study on Shanghai's Sports Event Policies**

| **Policy Name** | **Issue Date** | **Issuing Organization(s)** |
| --- | --- | --- |
| Notice on Issuing the “2022 Key Points of Shanghai Sports Industry Work” | 2022/3/21 | Shanghai Municipal Sports Bureau |
| 2021 Guidelines for Applying for Special Funds for the Development of Sports Events in Shanghai | 2022/1/12 | Shanghai Municipal Sports Bureau |
| Yangtze River Delta Sports Industry Integrated Development Plan (2021–2025) | 2022/1/7 | Sports Bureaus of Shanghai, Jiangsu, Zhejiang, Anhui |
| Opinions on Establishing a Long-Term Mechanism for Sports Event Safety Prevention | 2022/1/7 | Shanghai Municipal Sports Bureau |
| Several Provisions on Clarifying the Responsibilities of the Shanghai Sports Bureau in Sports Event Organization | 2022/1/4 | Shanghai Municipal Sports Bureau |
| Notice on the “14th Five-Year Plan for the Development of Shanghai’s Sports Industry” | 2021/12/1 | Shanghai Municipal Sports Bureau |
| Construction Plan for the Shanghai Sports Events System (2021–2025) | 2021/11/12 | Shanghai Municipal Sports Bureau |
| Application Guide for the “Shanghai Events” Brand Certification | 2021/11/2 | Shanghai Municipal Sports Bureau |
| Supplementary Notice on Strengthening the Safety Management of Sports Events | 2021/6/16 | Shanghai Municipal Sports Bureau |
| Guidelines for Hosting Sports Events During Regular Epidemic Prevention and Control (3rd Edition) | 2021/6/16 | Shanghai Municipal Sports Bureau |
| Notice on Further Strengthening the Safety Management of Sports Events and Activities | 2021/6/3 | Shanghai Municipal Sports Bureau |
| Notice on Issuing the “2021 Key Points of Shanghai Sports Industry Work” | 2021/4/2 | Shanghai Municipal Sports Bureau |
| 2020 Guidelines for the Second Batch of Special Fund Applications for Sports Event Development in Shanghai | 2020/12/15 | Shanghai Municipal Sports Bureau |
| Outline for Building Shanghai into a Globally Renowned Sports City | 2020/11/4 | General Office of the Municipal Government |
| Several Opinions on High-Quality Integrated Sports Development in the Yangtze River Delta | 2020/10/23 | Sports Bureaus of Shanghai, Jiangsu, Zhejiang, Anhui |
| Guidelines for Hosting Sports Events During Regular Epidemic Prevention and Control (2nd Edition) | 2020/9/24 | Shanghai Municipal Sports Bureau |
| 14th Five-Year Plan for the Development of Sports in Shanghai | 2020/9/23 | General Office of the Municipal Government |
| Notice on Guidelines for Hosting Sports Events During Regular Epidemic Prevention and Control | 2020/7/1 | Shanghai Municipal Sports Bureau |
| 2020 Guidelines for the First Batch of Special Fund Applications for Sports Event Development in Shanghai | 2020/4/21 | Shanghai Municipal Sports Bureau |
| 2020 Key Points of Shanghai Sports Industry Work | 2020/4/3 | Shanghai Municipal Sports Bureau |
| Administrative Measures for Sports Event Management in Shanghai | 2020/3/20 | Shanghai Municipal People’s Government |
| 2019 Guidelines for Special Fund Applications for Sports Event Development in Shanghai | 2019/7/23 | Shanghai Municipal Sports Bureau |
| Three-Year Action Plan for Building an International Sports Events Capital (2018–2020) | 2018/12/13 | Shanghai Municipal Sports Bureau |
| 2018 Guidelines for Special Fund Applications for Sports Event Development in Shanghai | 2018/8/22 | Shanghai Municipal Sports Bureau |
| Several Opinions on Accelerating Innovation in the Sports Industry in Shanghai | 2018/8/9 | Shanghai Municipal People’s Government |
| Interim Measures for the Construction and Management of Shanghai Sports Industry Clusters | 2018/9/7 | Shanghai Municipal Sports Bureau |
| 2018 Guidelines for Special Fund Applications for Sports Event Development in Shanghai | 2018/8/13 | Shanghai Municipal Sports Bureau |
| Provisions on the Organizational Structure of Sports Events in Shanghai (Trial) | 2018/7/16 | Shanghai Municipal Sports Bureau |
| Layout Plan for Sports Industry Clusters in Shanghai (2017–2020) | 2017/10/30 | Municipal Sports Bureau, Development and Reform Commission, Planning and Land Resources Bureau, Tourism Bureau |
| 2017 Guidelines for Special Fund Applications for Sports Event Development in Shanghai | 2017/9/1 | Shanghai Municipal Sports Bureau |
| Guiding Opinions on Hosting National Fitness Events in Shanghai | 2017/6/15 | Shanghai Municipal Sports Bureau |
| Implementation Plan for the Development of Shanghai’s Sports Industry (2016–2020) | 2017/2/14 | General Office of the Municipal Government |
| Notice on the 13th Five-Year Plan for Sports Reform and Development in Shanghai | 2016/12/12 | General Office of the Municipal Government |

**Table2. Core views from relevant experts on Shanghai's sports event development**

| **A: Core Experts** | **B: Core Perspectives on Promoting Shanghai Sports Events Development** |
| --- | --- |
| Haiyan Huang, Kaijuan Xu, Yin Li | Promote the collaborative hosting of regional events in the Yangtze River Delta region and jointly bid for major international sports events (Huang, 2021a; Li et al., 2020; Xu et al., 2019). |
| Lin Zhang, Nanzhu Li, Haiyan Huang, Kaijuan Xu, Yin Li | Strengthen information research and collection for sports events (Huang et al., 2007), establish a sports event information database (Li et al., 2020; Xu et al., 2019). |
| Lin Zhang, Nanzhu Li, Haiyan Huang, Qin Yao, Yinghui Zhang, Yin Li, Kaijuan Xu | Establish a development fund for major single-sport events (Huang et al. 2007), formulate special supportive policies for sports event development funding (Huang, 2019; Zhang et al., 2010; Zhang, 2015; Zhang & Huang, 2015). |
| Haiyan Huang, Yin Li | Improve sports event evaluation (Huang, 2021a), enhance assessment of events' contribution, professionalism, and public engagement (Li et al., 2020; Zhu et al., 2020). |
| Lin Zhang, Haiyan Huang, Yin Li | Shape Shanghai's sports event brand, cultivate local branded sports events (Huang, 2021a; Zhang, 2015), implement sports event brand certification (Li et al., 2020; Qin, 2021). |
| Lin Zhang, Haiyan Huang, Yin Li | Establish a Shanghai sports event system aligned with international event capitals (Huang, 2019; Huang, 2021a; Li et al., 2020; Zhang, 2015). |
| Haiyan Huang, Yin Li | Create sports event clusters, optimize spatial layout of sports events (Huang, 2019; Huang, 2021a; Li et al., 2020). |
| Haiyan Huang, Lin Xu, Lei Luo | Establish a diversified investment mechanism to promote social participation in event organization (Huang et al., 2013; Huang, 2016). |
| Haiyan Huang | Implement a "one-stop" service mechanism for sports events to reduce operational costs for market entities (Huang, 2021a; Gong, 2020). |
| Haiyan Huang | Strengthen market supervision of sports events (Huang, 2021a; Huang, 2021b). |

Note: The specific references in the table are as follows:

Gong, J. (2020, September 25). How can Shanghai sports enterprises enjoy the government's "one-stop" service? Experts guide. *Shangguan*. <https://export.shobserver.com/baijiahao/html/293814.html>

Huang, H. (2016). On constructing Shanghai into a world-famous sport city. *Sports Science Research*, 37(4), 11-13+21.

Huang, H. (2019). *Shanghai sports industry development report (2017-2018)*. Social Sciences Academic Press.

Huang, H. (2021a). Reflections and measures of building Shanghai into the capital of international sports events. *Sports Science Research*, 42(1), 8-14.

Huang, H. (2021b). *Shanghai sports industry development report (2019–2021)*. Social Sciences Academic Press.

Huang, H., Xu, L., Luo, L., et al. (2013). The interaction between sport events and tourism in Shanghai. *Journal of Shanghai University of Sport*, 37(5), 37-41+56. <https://10.16099/j.cnki.jsus.2013.05.014>.

Huang, H., Zhang, L., & Li, N. (2007). Study on the role of Shanghai government in the operation of major sports competition events. *Sports Science*, 27(2), 17-25. <https://10.16469/j.css.2007.02.003>.

Li, Y., Li, G., & Huang, H. (2020). Construction strategy of Shanghai sports event system from the perspective of global sports city. *Journal of Shanghai University of Sport*, 44(3), 17-26. <https://10.16099/j.sus.2020.03.002>

Qin, D. (2021, December 28). Shanghai pioneers "Shanghai Events" brand certification system. *Jiefang Daily*. <https://www.jfdaily.com.cn/news/detail?id=436571>

Xu, K., & Huang, H. (2019). The development trend, experience and development proposals of the sports industry in the Yangtze river delta. *China Sport Science and Technology*, 55(7), 45-55. <https://10.16470/j.csst.2019043>

Zhang, L. (2015). *Yangtze River Delta regional sports industry development report (2014-2015)*. Social Sciences Academic Press.

Zhang, L., & Huang, H. (2015). *Shanghai sports industry development report (2014-2015)*. Social Sciences Academic Press.

Zhang, L., Li, N., Yao, Q., et al. (2010). Research on the development orientation of Shanghai sports events. *Journal of Shanghai University of Sport*, 34(2), 11-15+27. <https://10.16099/j.cnki.jsus.2010.02.005>.

Zhu, H., & Xu, D. (2020, June 4). Hosting major events must give cities both "face" and "substance". *Xinhua News Agency New Media*. <https://baijiahao.baidu.com/s?id=1668548714765103707&wfr=spider&for=pc>

**Table 3. Proposals by Shanghai DPs on the Development of Sports Events in Shanghai**

| **Shanghai PD** | **Proposal Title** |
| --- | --- |
| Jian Wu | Proposal on Accelerating the Development of Shanghai as a "Global Esports Capital" and the Esports Industry (Proposal No. 0333 of the Fifth Session of the XVth Municipal People's Congress) |
| Yuan Li | Proposal on Shanghai Building a "Global Esports Capital" (Proposal No. 0489 of the Fifth Session of the XVth Municipal People's Congress) |
| Jian Wu | Proposal on Shanghai Promoting the Integrated Development of the Sports Industry in the Yangtze River Delta (Proposal No. 0283 of the Third Session of the XVth Municipal People's Congress) |
| Yuan Li | Proposal on Shanghai "Building a World-Renowned Sports City" (Proposal No. 0326 of the Second Session of the XVth Municipal People's Congress) |
| Sports Sector, Shanghai CPPCC(Chinese People's Political Consultative Conference) | Proposal on Stimulating Shanghai's Leading Role to Promote High-Quality Integrated Development of the Sports Industry in the Yangtze River Delta Region (Proposal No. 0595 of the Third Session of the XIIIth Municipal CPPCC) |
| Peihong Li, Xi Zang, Wei Jin, Xuefeng Jiang, Fang Li, Yanchun Cao, Nan Shao, Suo Qiu, Liping Zhang | Proposal on Strengthening Traditional Media Coverage for Building Shanghai into an International Sports Events Capital (Proposal No. 0667 of the Third Session of the XIIIth Municipal CPPCC) |
| Jin Pan, Boying Liu, Qingfeng Li, Ying Su, Jian Fei, Zhuoyun Yao | Proposal on Jointly Building the Sports Competition and Performance Industry in the Yangtze River Delta to Boost Shanghai's Construction as an International Sports Events Capital (Proposal No. 0653 of the Second Session of the XIIIth Municipal CPPCC) |
| Jingxuan Ma, Dingguo Gao, Yifa Chen, Shicheng Zhang, Yongkang Zhang, Yingyu Liang, Shan Jiang | Proposal on Building Shanghai into an International Esports Center (Proposal No. 0016 of the Second Session of the XIIIth Municipal CPPCC) |
| Yu Liu, Peijie Chen, Renwei Wang | Proposal on Seizing the National Strategy Opportunity of Yangtze River Delta Regional Integration to Solidly Advance the Integrated Development of the Sports Industry in the Yangtze River Delta Region (Proposal No. 0468 of the Second Session of the XIIIth Municipal CPPCC) |
| Depei Wang | Proposal on Introducing Rugby and Innovating Rugby (Proposal No. 0239 of the Fifth Session of the XIIth Municipal CPPCC) |
| Yimin Jin | Proposal on Developing Equestrian Sports to Assist in Building an Excellent Global City (Proposal No. 0127 of the Fifth Session of the XIIth Municipal CPPCC) |

**Table4. Sport Event-Hosting Policies Issued at the National Level in China (2016-2022)**

|  | Policy | Issue Date | Issuing Authority |
| --- | --- | --- | --- |
| 1 | Opinions on Further Strengthening Safety Supervision and Services for Sports Events | 2021/7/9 | General Administration of Sport of China et al. |
| 2 | Notice of the General Administration of Sport on Strengthening Safety Management of Road Running Events | 2021/6/2 | General Administration of Sport of China |
| 3 | Notice of the General Administration of Sport on Suspending Relevant Sports Activities | 2021/6/2 | General Administration of Sport of China |
| 4 | Several Opinions on Strengthening the Standardized Management of Conduct in Sports Venues (Jointly issued by GAS and MPS) | 2021/5/17 | General Administration of Sport of China |
| 5 | Administrative Measures for Sports Events (2020 Edition) | 2020/1/17 | General Administration of Sport of China |
| 6 | Outline of the Yangtze River Delta Regional Integration Development Plan | 2019/12/1 | State Council of China |

**Table5. Robustness testing**

| solution | Configurational analysis | raw  coverage | net  coverage | consistency |
| --- | --- | --- | --- | --- |
| Parsimonious | O/LCs | 0.0740741 | 0.0740741 | 1 |
|  | ~DPs*~SEMD | 0.814815 | 0.0740741 | 1 |
|  | ~NSEPs*~DPs | 0.851852 | 0.111111 | 1 |
|  | Solution coverage | 1 | | |
|  | Solution consistency | 1 | | |
| Intermediate | ~LGDGs*~O/LCs*~EPs*~DPs*SESCs*~SEMD | 0.222222 | 0.222222 | 1 |
|  | ~NSEPs*~LGDGs*~O/LCs*~EPs*~DPs*~SESCs*SEMD | 0.111111 | 0.111111 | 1 |
|  | ~NSEPs*LGDGs*~O/LCs*EPs*~DPs*~SESCs*~SEMD | 0.592593 | 0.592593 | 1 |
|  | NSEPs*LGDGs*O/LCs*EPs*DPs*~SESCs*~  SEMD | 0.0740741 | 0.0740741 | 1 |
|  | Solution coverage | 1 | | |
|  | Solution consistency | 1 | | |

Note:

NSEPs = National sports event policies;

LGDGs = Local government development goals;

O/LCs = Organizational reforms or changes in leadership;

Eps = Experts' perspectives

DPs = Proposals from deputies of the Shanghai Municipal People’s Congress

SESCs = Sport event safety crises

SEMD = Sport event market disorganization
